# Topological beaming of light: proof-of-concept experiment

**DOI:** 10.1038/s41377-025-01799-w

**Published:** 2025-03-13

**Authors:** Yu Sung Choi, Ki Young Lee, Soo-Chan An, Minchul Jang, Youngjae Kim, Seungjin Yoon, Seung Han Shin, Jae Woong Yoon

**Affiliations:** 1 Department of Physics, Hanyang University, Seoul, 133-791 South Korea; 2 Convergence Technology Division, Korea Advanced Nano Fab Center, Suwon, 16229 South Korea; 3 Joint Quantum Institute, University of Maryland, College Park, MD 20742 USA

**Keywords:** Nanophotonics and plasmonics, Photonic devices

## Abstract

Beam shaping in nanophotonic systems remains a challenge due to the reliance on complex heuristic optimization procedures. In this work, we experimentally demonstrate a novel approach to topological beam shaping using Jackiw-Rebbi states in metasurfaces. By fabricating thin-film dielectric structures with engineered Dirac-mass distributions, we create domain walls that allow precise control over beam profiles. We observe the emergence of Jackiw-Rebbi states and confirm their localized characteristics. Notably, we achieve a flat-top beam profile by carefully tailoring the Dirac-mass distribution, highlighting the potential of this method for customized beam shaping. This experimental realization establishes our approach as a new mechanism for beam control, rooted in topological physics, and offers an efficient strategy for nanophotonic design.

## Abstract (web summary)

Topological beam shaping using Jackiw-Rebbi states enables precise control of beam profiles in nanophotonic systems.

## Graphical abstract

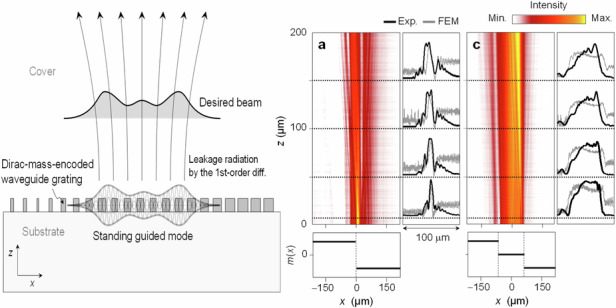

## Introduction

Precise control and shaping of light beams are crucial for a wide range of applications, including laser machining, laser therapy, optical communications, and emerging quantum technologies^1–9^. Traditionally, beam shaping has relied on optical elements such as refractive and diffractive optical elements (DOEs)^10–14^ and spatial light modulators^15–17^. Although these methods are effective, they generally involve heuristic optimization algorithms, substantially limiting their adaptability particularly for intricate nanophotonic structures.

Recent advances in topological photonics have opened new avenues for manipulating light in unprecedented ways^18–21^. Among various intriguing effects, the Jackiw-Rebbi (JR) soliton, a zero-energy solution for a domain-wall Dirac equation, attracts considerable interest because of its significance in fundamental physics of solid-state systems and topological photonic device applications^22–34^. Toward this end, topological beam-shaping method was theoretically proposed as a promising design principle^35^. In this method, a photonic analogy of the JR state and its new degree of control freedom enables a remarkably efficient beam shaping in a systematic manner that does not require tedious optimization steps.

Here, we present the first experimental demonstration of the topological beam-shaping principle. In our original proposal^35^, we theoretically confirmed that Bragg-reflection rate of a guided-mode resonance (GMR) plays an identical role of mass of a Dirac fermion in its wave-kinematic property which leads to band topology identical to 1D topological insulators. On this basis, a GMR analogy of a JR soliton enables efficient shaping of its leakage-radiation beam by means of a continuous modulation in the Bragg-reflection rate. In this paper, we experimentally realize this beam shaping GMR structure in lossless dielectric media in the optical telecommunications band. The fabricated devices reveal key spectral and spatial properties of the prescribed GMR JR state and indeed produce the desired flat-top beams in experiments. We discuss the inherent limitations of our experimental results and propose a potential solution based on monolithic geometry control of the unit-cell design.

## Results

### Theory

**Fig. 1:**
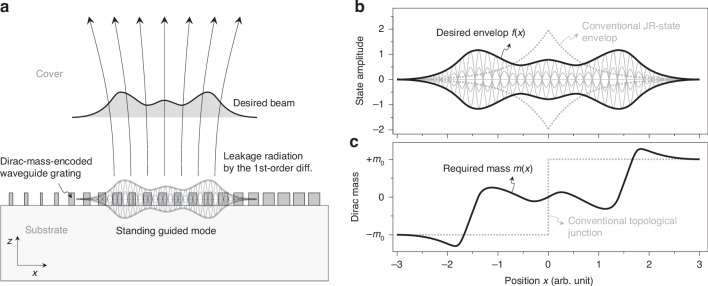
**Principles of topological beam shaping and Jackiw-Rebbi state engineering in metasurfaces**. **a** Schematic representation of leakage-radiation beam shaping with engineered Dirac mass distribution. The guided-mode resonance (GMR) with a tailored standing-wave envelope emits a desired beam profile through first-order diffraction. **b** Characteristic bi-exponential envelope profile *f*(*x*) of the Jackiw-Rebbi (JR) state at a topological junction (gray dotted curve). **c** Corresponding spatially-varying Dirac mass distribution *m*(*x*) that generates the JR state profile in (**b**)

The leakage-radiation beam shaping here is schematically illustrated in Fig. 1a. A waveguide grating with carefully designed unit-cell structure supports a leaky guided modes with a prescribed standing-wave envelope profile. A desired beam of leakage radiation is emitted by the first-order diffraction from the guided-mode fields as it transfers the standing-wave envelop profile in the guided mode to the emitted beam.

Previously, obtaining an appropriate waveguide-grating design for a certain desired standing-wave envelope in the guided-mode field is nontrivial and generally involves trial-solution-based numerical optimization algorithms in the similar manner as conventional beam-shaping DOEs. Intriguingly, our previous work^35^ theoretically suggests a simple rule to configure a guided-mode standing-wave envelope profile as we explain hereafter.

The proposed approach is based on continuous tuning of coupling strength between the left-going and right-going guided modes at their second-order Bragg reflection condition. Let us represent amplitudes of the left-going and right-going guided-mode amplitudes by *ψ*_L_ and *ψ*_R_, respectively. Coupling constant between *ψ*_L_ and *ψ*_R_ is denoted by *κ* which is basically an amplitude of the Bragg reflection of the guided modes through the second-order diffraction processes. *κ* is quantitatively defined as indicating the second-order Bragg-reflection rate. In the approach proposed in ref. ^35^, *κ* is assumed to be a slowly varying function of position *x* along the axis of periodicity and its position dependence is locally related to the standing guided-mode envelope function *f*(*x*) by

1 $$\kappa (x)=-c\frac{1}{f(x)}\frac{df(x)}{dx}$$

where *c* is speed of light in vacuum. Equation (1) tells a required *κ*(*x*) distribution for obtaining a certain desired envelope *f*(*x*) profile. Once a desired *f*(*x*) is induced by an appropriately configured *κ*(*x*) from Eq. (1), *f*(*x*) is directly transferred to a beam of leakage radiation emitted from the standing guided mode through the first-order diffraction process. The fundamental principle leading to Eq. (1) is derived from the mathematical analogy of GMRs near the second-order Bragg condition to the 1D topological insulator and equivalently the 1D Dirac equation, as explained in detail in ref. ^35^.

Briefly describing the key concept, *κ* in a GMR system formally corresponds to mass *m* of a 1D Dirac fermion and also the difference *w*−*v* between inter-cell (*w*) and intra-cell (*v*) coupling potentials in a dimerized atomic chain known as the Su-Schrieffer-Heeger (SSH) model such that

2 $$\hslash \kappa =m{c}^{2}=w-v$$

This parametric correspondence immediately implies that band topology described by the parameter *w*−*v* in the SSH model appears in the Dirac fermion and GMR eigensystems in the same manner in terms of the Dirac mass *m* and Bragg-reflection rate *κ*, respectively. Note that ℏ in Eq. (2) does not indicate our case here is a sort of quantum systems. ℏ is included just for the parametric correspondence to be in the proper dimension and unit system. Therefore, Eq. (2) simply implies that classical electromagnetic-field properties of our GMR system simulates wave-kinematic properties of quantum systems for the Dirac Fermions and SSH model. In particular, the topologically trivial phase is characterized by *κ* > 0 (*m* > 0 and *w*−*v* > 0 equivalently) with topological invariants of the winding number 0 and Zak phase 0, while the topologically non-trivial phase is characterized by *κ* < 0 (*m* < 0 and *w*−*v* > 0 equivalently) with the winding number 1 and Zak phase π. These two distinguished topological phases in general cannot transit from and to each other without nonadiabatic changes including a bandgap closing at the critical phase for *κ* = *m* = *w*−*v* = 0 as known well in the 1D topological insulator theory ^36,37^.

Subsequently, a GMR topological junction is created between two adjacent GMR structures with *κ* > 0 on one side and *κ* < 0 on the other side. A topological edge state at this junction is in direct mathematical analogy to the JR soliton for a localized Dirac fermion state with the linear *κ*-*m* relation in Eq. (2). Therefore, a localized GMR state at the junction naturally inherits all properties of the JR soliton state including its relation between localization profile *f*(*x*) and Dirac mass distribution *m*(*x*), i.e., *mc*^2^ = −(*ħc*)·*f*^−1^*df*/*dx*, which leads to Eq. (1) for the GMR state. This relation describes a JR state as shown in Fig. 1b, c. The gray-dotted curves schematically show typical a bi-exponential *f*(*x*) for a piecewise-constant *m*(*x*) distribution about the junction. If we modify the *m*(*x*) distribution to a certain continuous function containing at least one or more junction points at which sign of *m* changes, *f*(*x*) can take any arbitrary shape in principle, as indicated by the solid curves. The identical *f* shaping strategy is also possible for GMR states with the *κ*-*m* correspondence in Eq. (2). See [refs. ^25,35–37^] for detailed mathematical treatment.

We note that the analogy between a GMR and Dirac fermion is physically stronger than the analogy between SSH model analogy. The connection of the SSH model to the Dirac fermion in Eq. (2) requires the low-energy continuum approximation in order to interpret spatially discretized tight-binding amplitudes in the SSH model as a continuous wave function of a Dirac fermion. However, the GMR-Dirac-fermion connection does not require such approximation because the guided mode wave function is continuous over the space by its nature. Therefore, the proposed GMR analogy to the JR state is valid as far as the coupled-mode theory of GMR leading to Eq. (2) is physically reasonable. According to our previous paper^25^, the coupled-mode theory is based on an Ansatz of the total-field expression which consists of dominant zero-order harmonic in the optical far field and ±1-order harmonics in the optical near field in the form of guided modes. This assumption is valid for most GMR structures with moderate index contrast in the order of 1.

Further explaining our approach based on Eq. (1) in structure-design perspective, we consider a waveguide grating structure with a single ridge per unit cell. In such a case, the second-order Bragg-reflection rate takes an expression as

3 $$\kappa =[{D}_{1}\,{F}^{2}{\Delta} {\varepsilon }^{2}{{\rm{sinc}}}^{2}(F)-{D}_{2}\,F{\Delta} \varepsilon \,{\rm{sinc}}(2F)]{\omega }_{0}$$

where Δ*ε* is dielectric-constant difference between the high-index and low-index parts of the grating, fill factor *F* is relative width of the grating ridge to the period, and *ω*_0_ is frequency of the second-order Bragg-reflection for the guided mode. *D*_*m*_ is diffraction-strength constant which is determined by the following relations.

4 $${D}_{1}=\frac{{n}_{{\rm{p}}}}{2{n}_{{\rm{g}}}}\mathop{\iint }\limits_{\begin{array}{c}{\rm{Grating}}\\ {\rm{layer}}\end{array}}dz\,dz^{\prime}\,{{k}_{0}}^{2}\,{u}^{\ast }(z)\,G(z,z^{\prime})\,u(z^{\prime})$$

5 $${D}_{2}=\frac{{n}_{{\rm{p}}}}{2{n}_{{\rm{g}}}}\mathop{\int }\limits_{\begin{array}{c}{\rm{Grating}}\\ {\rm{layer}}\end{array}}dz\,|u(z){|}^{2}$$

where *n*_p_ and *n*_g_ denote phase and group speed indices, respectively, *k*_0_ is vacuum wave number, *u*(*z*) is normalized wave function of the guided mode, and *G*(*z*,*z*′) is 1D Green’s function for an effective 1D structure that the grating layer is replaced by a homogeneous effective medium. We note that *D*_1_ denotes strength of two consecutive first-order diffractions of the guided mode in the grating layer while *D*_2_ indicates strength of the single second-order diffraction. See [refs. ^25,35^] for details of the theory leading to these relations. Combining Eqs. (1) and (3), we obtain a simple mapping between desired envelope profile *f*(*x*) and fill-factor distribution *F*(*x*) such that

6 $$[{D}_{1}{F}^{2}{\Delta} \varepsilon \,{{\rm{sinc}}}^{2}(F)-{D}_{2}F\,{\rm{sinc}}(2F)]=-{\Delta} {\varepsilon }^{-1}\frac{1}{f}\frac{df}{{k}_{0}dx}$$

### Experiment: Basic spectral properties

**Fig. 2:**
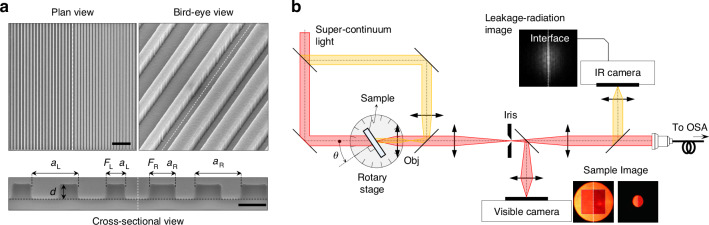
**Experimental realization of topological Jackiw-Rebbi state**. **a** Focused ion beam image of the fabricated topological junction device from plan (scale bar, 5 μm), bird-eye and cross-sectional (scale bar, 500 nm) views. **b** Schematic of the angle-resolved confocal microscopy setup. Obj, objective lens; OSA, optical spectrum analyzer. Inset images display the sample (obtained from visible camera) and radiation pattern (from IR camera), with white dashed lines marking the topological interface

To verify our theoretical proposal in experiments, we fabricate GMR-structure samples using standard e-beam lithography. Briefly, a silicon-nitride (SiN) layer with thickness of 300 nm is first deposited on the quartz wafer as the slab waveguide layer. Then, the SiN grating patterns are produced using e-beam lithography and etching process. A 2-μm-thick SiO_2_ cover layer is deposited. The focused ion beam images for a topological junction sample are shown in Fig. 2a. The left and right sides of the junction have different fill-factors *F* and periods *a*. In this case, we chose *F*_L_ = 0.3, *a*_L_ = 880 nm for the left side and *F*_R_ = 0.6, *a*_R_ = 840 nm for the right side. See “Materials and Methods” for details of the fabrication steps and conditions.

In spectral measurement of the sample for the desired topological properties, we use a microscopic angle-resolved spectrum analysis set up, as schematically illustrated in Fig. 2b. The set-up acquires angle-resolved transmittance spectrum at a designated microscopic spot on the sample, enabling position-dependent spectrum analysis. A collimated supercontinuum light beam (highlighted in red) is incident on the sample at angle *θ*. The transmitted beam is collected by the objective lens. The tube lens forms a magnified image of the sample. An iris-diaphragm at the magnified-image plane designates the measurement spot on the sample. The image of the selected spot is acquired with the visible-image sensor on the bottom in order to conveniently adjust size and location of the measurement spot. Meanwhile, part of the transmitted light is coupled to a fiber connected to an optical spectrum analyzer. The parts associated with the beams highlighted in yellow are for leakage-radiation beam analysis that will be explained in the next section.

**Fig. 3:**
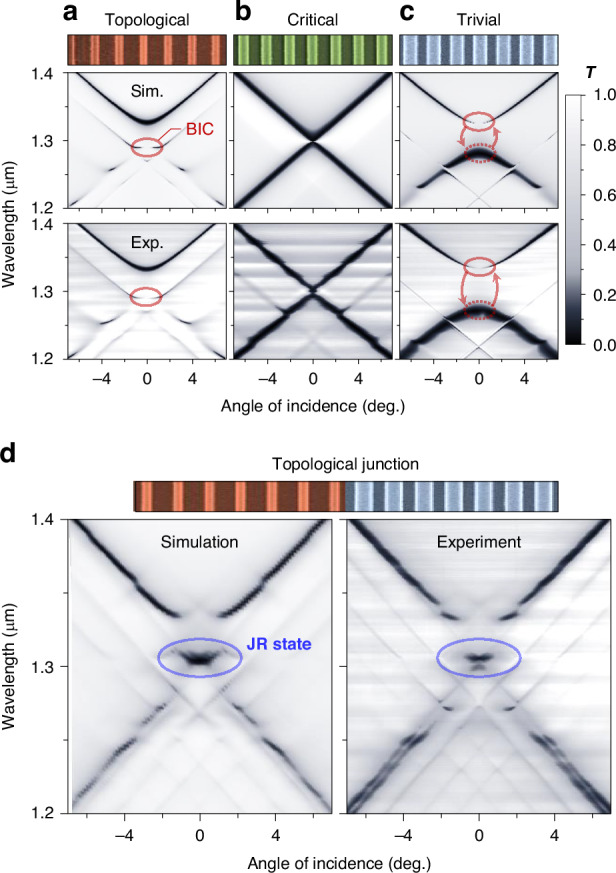
**Angle-resolved transmission spectra demonstrating topological phase transition and emergence of the Jackiw-Rebbi state**. **a** Topological phase, *F* = 0.3, *a* = 880 nm. **b** Critical phase, *F* = 0.44, *a* = 860 nm. **c** Trivial phase, *F* = 0.6, *a* = 840 nm. **d** Topological junction sample, revealing a localized JR state within the bandgap

Angle-resolved transmittance spectra of our fabricated samples reveal definite evidence for the topological phase transition and emergence of the photonic JR state. In Fig. 3, we show experimental spectra in excellent agreement with the rigorous numerical simulation by the finite-element method (FEM). Figure 3a–c experimentally confirm the topological phase transition with the fill-factor change. A characteristic feature of the topological phase transition is a spectral flip of dark and bright resonance band edges. We indicate the dark resonance, often referred to as bound state in the continuum (BIC), with red ellipse in Fig. 3a for the topological phase for *F* = 0.3. The BIC is at the lower-wavelength band edge therein. As *F* increases to 0.44, the GMR is at the topologically critical phase and Dirac-point-like resonance-band crossing appears, as shown in Fig. 3b. As *F* increases further, the band gap opens again and the BIC is now at the upper-wavelength band edge, as shown in Fig. 3c for *F* = 0.6.

Of particular interest in our study here is the JR-state resonance for topological junction structures. In Fig. 3d, we show the spectrum for the junction sample consisting of two topologically distinguished structures in Fig. 3a, c. The experimental spectrum contains a clear signature of the JR-state resonance at the center (~1.3 μm) of the band gap again in excellent agreement with the numerical simulation. In further detail, this JR-state resonance corresponds to the case illustrated in Fig. 1b, c for the conventional JR-state envelope. Thereby, it takes a bi-exponential decaying envelope profile localized at the junction interface. Although the observed JR-state feature in the spectral domain is a definite evidence, more direct observation of its localization property should be further investigated to completely reveal its unique characteristics in experiment.

**Fig. 4:**
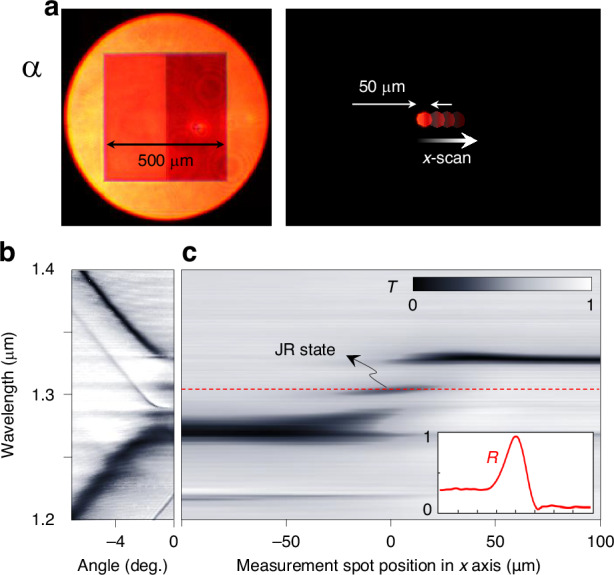
**Spatial characteristics of the Jackiw-Rebbi state**. **a** Visible camera images of the sample and measurement area. **b** Angle-resolved transmission spectrum of the junction structure. **c** Local resonance-spectrum under normal incidence with inset showing reflectance profile at the JR resonance wavelength (red dashed)

Consequently, we perform local spectral analysis by taking measurement aperture size at its our technical minimum ~50 μm, as shown in Fig. 4a. We acquire normal-incidence transmittance spectrum while the 50-μm-wide spot scans its *x*-position across the topological-junction interface. The measured spectra are summarized in Fig. 4b, c. In Fig. 4b, we show the angle-resolved spectrum when the spot is exactly at the junction interface for reference. The JR-state resonance feature is at wavelength 1.3 μm and incident angle 0. The *x*-scan normal-incidence spectrum in Fig. 4c confirms that the JR-state resonance is localized to the junction interface as the resonance feature is excited only when the spot is in close vicinity of the junction interface. This localization property is in stark contrast to the spatially uniform band-edge resonance features at 1.27 and 1.33 μm.

Full-width at half maximum of the JR-state feature in the *x*-scan data is 29 μm, which is substantially larger than the theoretical JR-state envelope width ~12 μm. This width broadening is attributed to boxcar-average blurring due to the 50-μm-wide aperture. Although significant blurring effect exists in the *x*-scan spectral data, it approximates the JR-state envelope profile as indicated in the inset of Fig. 4c. Therein, we indicate the *x*-scan reflectance spectrum at the JR-state resonance-center wavelength (red-dashed line).

### Experiment: Leakage-radiation beam shaping

As discussed in the theory section, the leakage-radiation distribution from the guided-mode JR state can be adaptably shaped into any desired form by the Bragg-reflection-rate mapping. In this section, we investigate this intriguing possibility in experiment.

**Fig. 5:**
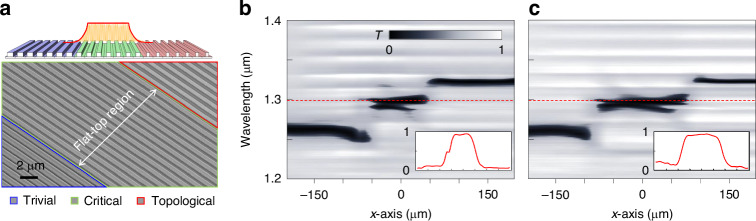
**Experimental realization of flat-top beam shaping**. **a** Schematic overview of the structure designed for flat-top beam profile generation, illustrating trivial (blue), critical (green), and topological (red) phases. Scale bar, 2 μm. **b**, **c** Local transmission spectra for the junction structure with flat-top regions of widths 85 μm and 127.5 μm, respectively. Insets display the reflectance spectra at the JR state resonance wavelength (red dashed line, 1.3 μm), demonstrating the achieved flat-top beam profiles

We fabricate a specific junction structure that creates a flat-top beam, as shown in Fig. 5a. Such junction consists of three piece-wise-constant regions of *m*(*x* < −*w*/2) = +*m*_0_ (trivial phase), *m*(−*w*/2 ≤ *x* ≤ +*w*/2) = 0 (critical phase), and *m*(*x* > +*w*/2) = −*m*_0_ (topological phase), respectively. These regions are indicated by blue, green, and red highlights in Fig. 5a for the design schematic on top and actual fabricated sample on the bottom.

For fabricated samples with *w* = 85 and 127.5 μm, we perform the local resonance-spectrum analysis identical to Fig. 4c, as shown in Fig. 5b, c, respectively. We see clear JR-state resonance features at 1.3 μm wavelength, which are elongated in *x*-axis over domains consistent with applied *w* values. The reflectance spatial profiles at 1.3 μm wavelength is provided in the insets and they show favorably flat-top profiles as expected.

Therefore, we presume that the JR-states in these samples have flat-top envelope profiles at the desired width values.

Now, we observe leakage-radiation distribution from the JR-state resonance. In our passive GMR structure case, directly observing pure leakage radiation is quite challenging because there are no emissive or florescent elements that excite the resonance state in the absence of light incidence at the resonance wavelength. Consequently, we take a passive mode-filtering technique. A sufficiently wide and collimated light beam is incident on the sample at *θ* = 0 and reflected beam distribution on *x*-*z* plane at the resonance wavelength is acquired by an IR camera scanning its object plane from the sample surface (*z* = 0) to a certain prescribed distance (*z* = *L*). This measurement is done with the set-up in Fig. 2b by incorporating the additional beam paths highlighted in yellow and their pertaining components. We take two reflected beam distributions *U*_s_(*x*, *z*) and *U*_ref_(*x*, *z*) separately from the topological-junction sample and unpatterned reference area, respectively. Finally, we take *U*_LR_(*x*, *z*) = *U*_s_(*x*, *z*)−*U*_ref_(*x*, *z*) as an approximate leakage-radiation distribution. This method is based on general property of GMR that the reflected light *U*_s_ can be decomposed by dominant leakage radiation *U*_LR_ part from the resonance state and weak non-resonant zero-order reflection *U*_0_ part. Since *U*_ref_ from the unpatterned area approximates *U*_0_ in our moderate index-contrast structure, we can infer *U*_LR_ as *U*_LR_(*x*, *z*) = *U*_s_(*x*, *z*)−*U*_0_(*x*, *z*) ≈ *U*_s_(*x*, *z*)−*U*_ref_(*x*, *z*).

**Fig. 6:**
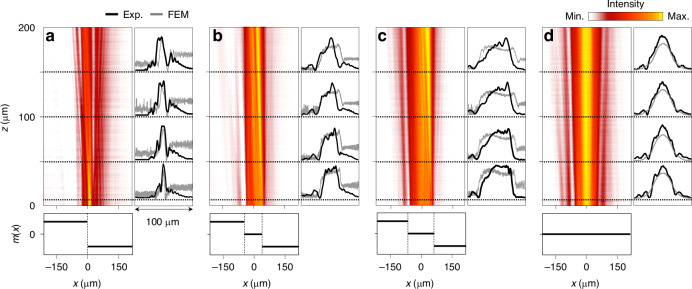
**Measured leakage-radiation distributions** ***U***_LR_(x, z) **for various Dirac mass configurations.** **a**–**c** Topological junction structure with critical phase widths *w* of 0, 85 μm, 127.5 μm, respectively. **d** Conventional GMR structure with constant Dirac mass (*w* = 500 μm). Left panels show 2D distributions over |*x*| ≤ 200 μm and 0 ≤ *z* ≤ 200 μm. Right graphs compare experimental beam profiles (black lines) with FEM simulations (gray lines). Bottom panels illustrate corresponding Dirac mass distributions *m*(*x*) over the measurement region

The measurement result is summarized in Fig. 6. We present experimentally inferred *U*_LR_(*x*, 0 ≤ *z* ≤ 200 μm) over a domain |*x*| ≤ 200 μm and 0 ≤ *z* ≤ 200 μm for four selected *m*(*x*) conditions of *w* = 0, 85 μm, 127.5 μm, and 500 μm under Gaussian beam incidence with diameter 80 μm. For each case, we compare the experimental beam profiles on four selected planes at *z* = 5, 50, 100, 150 μm with rigorous numerical calculation due to the FEM. We confirm good agreement between the experiment and simulation for all four *m*(*x*) conditions. In particular, the leakage-radiation beam width increases with increasing junction-domain width *w* from 0 to 127.5 μm, as seen from Fig. 6a–c. The case for *w* = 127.5 μm in Fig. 6c exemplifies the strong effect of the JR-state resonance because flat-top-like beam profile is observed even though the junction width is considerably wider than 80 μm for the incident beam width.

The flat-top-like beam profile is clearly due to the characteristic effect of the topological junction because a conventional GMR structure does not have such property. In Fig. 6d for *w* = 500 μm, the leakage-radiation beam takes a Gaussian profile which is simply identical to the incident beam. In this case, the entire sample domain with footprint width 500 μm has a constant Dirac mass at *m*(*x*) = 0 with no junction structure.

## Discussion

Although the results in Fig. 6 confirm remarkable effect of the topological junction structure on the leakage-radiation beam properties, neither the experimental nor numerical beam shapes precisely show the desired flat-top profiles. This issue should be properly addressed because accurate generation of a desired beam profile is crucial in consideration of practical applications.

We attribute the observed inaccuracy to inhomogeneity of first-order diffraction amplitude across the junction region. In Fig. 6, an apparent error is the asymmetric beam shape which is certainly not a feature of the JR-state envelope. Assuming that JR-state envelope *f*(*x*) is symmetric with respect to the junction center at *x* = 0 as intended from the structure design, the only factor that can possibly produce the strong asymmetry in the emitted beam is uneven distribution of first-order diffraction amplitude. Although it was not pointed out in our original theoretical proposal^35^, uniform first-order diffraction amplitude is a necessary requirement for the precise beam shaping.

In further detail, we treat the leakage radiation wavefunction *L*(*x*,*z*) based on the coupled-mode model developed by Kazarinov and Henry^38–41^. The model describes as

7 $$L(x,z)={\varepsilon }_{1}(x)({\psi }_{{\rm{L}}}+{\psi }_{{\rm{R}}})W(z)$$

8 $$W(z)=-{{k}_{0}}^{2}\mathop{\int }\limits_{\begin{array}{c}{\rm{Grating}}\\ {\rm{layer}}\end{array}}dz^{\prime}\,G(z,z^{\prime})u(z^{\prime})$$

where *ε*_1_(*x*) is a first-order harmonic amplitude of the periodic dielectric function in the grating layer whose unit-cell design is slowly varying in *x*, *ψ*_L_ and *ψ*_R_ denote left-going (L) and right-going (R) guided-mode amplitudes, and *W*(*z*) is normalized leakage-radiation wave function in *z*-axis. Combining Eq. (7) with the standard JR-state solution [*ψ*_L_, *ψ*_R_] = 2^1/2^[1−*i*, 1 + *i*], we obtain an approximate leakage-radiation wave function *L*_JR_ as

9 $${L}_{{\rm{JR}}}(x,z)\approx {\varepsilon }_{1}(x)f(x)\,W(z)$$

Although a complete description of *L*_JR_ with non-uniform excitation of guided modes requires a weighted superposition of *L*’s for different *k* components taking Fourier transform *F*(*k*) of *f*(*x*) as the weight, Eq. (9) should provide a reasonable approximation for slowly-varying envelope cases. As clearly revealed in Eq. (9), the leakage-radiation profile is *ε*_1_(*x*)·*f*(*x*), not *f*(*x*). Consequently, local first-order Fourier coefficient *ε*_1_(*x*) has to be constant in *x* in order to produce an exact replica of *f*(*x*) in beam profile *L*_JR_.

Unfortunately, keeping *ε*_1_(*x*) constant for the precise beam shaping seems impossible when we consider that certain non-uniform *ε*_1_(*x*) distribution is inevitable for synthesizing required *κ*(*x*) distribution according to Eqs. (3) and (6). Following the mathematical treatment in [refs. ^25,35^], Eq. (3) for the second-order Bragg-reflection rate can be alternatively expressed in terms of Fourier transform *ε*_*m*_ of the dielectric function in the grating layer as

10 $$\kappa =({D}_{1}\,{{\varepsilon }_{1}}^{2}-{D}_{2}{\varepsilon }_{2}){\omega }_{0}$$

This relation implies that we have to be able to change *ε*_2_ without altering *ε*_1_ for synthesizing a required *κ* distribution with constant *ε*_1_ distribution. For single-ridge unit-cell structures, fill factor *F* is the only factor that can be used to tune *κ* and it simultaneously changes both *ε*_1_ and *ε*_2_ because *ε*_1_ = *F*Δ*ε* sinc(*F*) and *ε*_2_ = *F*Δ*ε* sinc(2 *F*). Therefore, the precise beam shaping with a single-ridge unit-cell structure is in principle impossible, significantly restricting application potential of our Dirac-mass-control approach.

However, changing *ε*_2_ without altering *ε*_1_ may become readily possible if we break the grating ridge into multiple parts within the unit cell. In such compound-grating structures, relative widths and positions of multiple parts can be used as extra degrees of design freedom, which allow various schemes for tuning *ε*_2_ under the constraint of constant *ε*_1_. For example, let us consider a grating unit cell consisting of two grating ridges specified by their interval Δ and fill factors *F*_1_ and *F*_2_. In such structures, the Fourier harmonic amplitude *ε*_*m*_ is described as a function in a three-dimensional parametric space (*F*_1_, *F*_2_, Δ). We can envisage a two-dimensional parametric subspace specified by an equation *ε*_1_(*F*_1_, *F*_2_, Δ) = *constant*. On this subspace, *ε*_2_(*F*_1_, *F*_2_, Δ) in general must be variable within a certain range. Thereby, we can in principle use this parametric subspace as an available design parametric space for obtaining desired *κ* distribution while keeping *ε*_1_ constant. Nevertheless, there might be some cases where the accessible range of *κ* values over the parametric subspace is not enough for a desired beam profile. In such cases, one can expand the parametric subspace for the required *κ*-range enhancement by introducing additional parametric dimensions such as thicknesses of the grating, waveguide, and additional layers.

The Dirac-mass modulation for leakage-radiation beam shaping can be used as an effective degree of control freedom for applications to surface emitting lasers^42–44^. For example, Dirac-singularity cavities^42^ were proposed for scale-invariant single-mode laser operation. In this proposal, high-order transversal modes in a remarkably large cavity are efficiently suppressed in the presence of lateral radiation loss which is unfavorable for practical applications. Our proposed approach may improve surface-emitting laser performance within this context because a JR-state GMR does not necessarily involve lateral radiation loss and associated degradation in the resonance quality factor while emitted beam profile and subsequent angular distribution in the optical far field are efficiently controllable. In a broader perspective, mode shaping based on continuous topological-parameter modulation can be applied for other purposes such as topological channel guided-mode shaping^45^ and efficient critical coupling of resonances with nonideal laser beams ^46^.

In conclusion, we have successfully demonstrated the topological beam-shaping approach through experimental validation. By leveraging the wave-kinematic analogy between guided modes and 1D Dirac fermions, we designed and fabricated a topological-junction structure that supports a guided-mode standing-wave field and its leakage radiation with the desired envelope distribution. Experimental data, obtained through angle-resolved local spectrum analysis and passive beam-profiling method, show excellent agreement with theoretical predictions. However, the results also highlight a limitation in the precision of the generated beam shape. We identify a primary cause of this limitation and propose a potential solution involving compound unit-cell structures, which avoids the need for additional materials or fabrication steps. The compound unit-cell structure is particularly promising for future research, as it not only has the potential to enhance beam shape accuracy but also may facilitate the creation of higher-dimensional topological phases with greater design flexibility. These advancements are crucial for practical applications of topological physics.

## Materials and methods

### Numerical simulation details

All numerical simulations here are performed using a commercial FEM solver (COMSOL Multiphysics 5.5). Two-dimensional models (in the x-y plane) are created to simulate one-dimensional photonic lattices with perfectly matched layers along the y-direction. The scattering and periodic (Floquet) boundary conditions are applied along the x-direction and y-direction, respectively. The junction structure was simulated for guided mode resonance lattices with trivial and topological phases, each composed of 20-unit cells in a supercell arrangement. The transmission spectra in Fig. 3 are calculated for TE-polarized plane waves at incident angles ranging from −7° to 7°, with measurements taken from the output port.

### Fabrication

The sample is fabricated on a double-side polished quartz wafer with a silicon nitride layer thickness of 300 nm, and a silica substrate of thickness around 500 μm. The step-by-step fabrication process is illustrated in Supplementary Fig. S1. A 300 nm thick SiN film is thermally deposited using plasma-enhanced chemical vapor deposition (PECVD). A layer of ZEP520A e-beam resist (300 nm thick) is spin-coated on the SiN layer. The one-dimensional photonic lattice pattern is then defined using electron-beam lithography (JBX 9300-FS). The photonic patterns are defined in the photo-resist layer, which is then developed in pentyl acetate (ZED-N50) for 90 s. The SiN layer with the resist pattern is etched in an inductively coupled plasma-reactive ion etcher with SF_6_ + O_2_ gas mixture. After residual PR removal, a 2 μm thick SiO_2_ cover is deposited using PECVD. Finally, the fabricated sample on the 4” quartz wafer is cleaved into 2 cm × 2 cm chips and cleaned. See Supplementary Materials for further details of fabrication steps and conditions.

## Supplementary information

Supplementary information

## Data Availability

All data needed to evaluate the conclusions in the paper are present in the paper and/or the Supplementary Materials.
