# Supplementary material for: Topological beaming of light: proof-of-concept experiment
Source: Light Sci Appl. 2025 Mar 13;14:121. doi: 10.1038/s41377-025-01799-w (PMC11904235; doi:10.1038/s41377-025-01799-w)
Supplement: Supplementary file 1 — Supplementary information [file 41377_2025_1799_MOESM1_ESM.docx]

Supplementary information for:

**Topological beaming of light: Proof-of-concept experiment**

Yu Sung Choi^1,†^, Ki Young Lee^1,†^, Soo-Chan An^1^, Minchul Jang^2^, Youngjae Kim^2^, Seungjin Yoon^3^, Seung Han Shin^1^, and Jae Woong Yoon^1,*^

*^1^Department of Physics, Hanyang University, Seoul, 133-791, Korea*

*^2^Convergence Technology Division, Korea Advanced Nano Fab Center, Suwon 16229, Korea*

*^3^Joint Quantum Institute, University of Maryland, College Park, MD 20742, USA*

^†^*These authors contributed equally to this work.*

^*^*Corresponding authors: yoonjw@hanyang.ac.kr*

**Sample fabrication details**

To fabricate the topological junction structure for validating our theoretical findings, we employ photolithography and E-beam lithography techniques. The complete process flow schematic for device fabrication is illustrated in Fig. S1. For the E-beam lithography process, Alignment markers and Chip lines necessary for component placement were produced using photo lithography. The Alignment marker consists of global and local keys, each with dimensions around 4 μm in width and 50 μm in length. Negative resist (DNR L300-D1, DONGJIN, KOR) with a thickness of 2 μm is coated on a Quartz wafer. Using an i-line stepper (NSR-2005i10C, Nikon), the patterns of the markers and keys engraved on the mask are exposed onto the wafer. The exposed chips undergo post-exposure bake (PEB) at 100 °C before development. To enhance the adhesive properties of the metal on the pattern surface, surface treatment is conducted using O2 gas. Subsequently, Cr 10 nm and Au 200 nm are deposited using an E-beam evaporator (EI-5, ULVAC). Finally, the completion s of the align pattern for E-beam lithography is achieved through the metal lift-off process using acetone and IPA solutions.

The designed device consists of various periods and structural parameters, so we proceed with the meticulous E-beam lithography process as follows. In e-beam lithography, the area that can be exposed without moving the stage and by maximally deflecting the electron beam is called the working field. If the continuous pattern exceeds the working field, Stitching phenomena can occur. Considering these phenomena, the device area is set to 500 × 500 μm^2^. A SiN layer of 300 nm is deposited on the sample with completed align key pattern using PECVD (P500, Applied Materials). Next, after plasma treatment, a positive resist (ZEP520A) is coated to a thickness of 300 nm. Since a Quartz substrate is used in this process, an additional conductive layer step is required to prevent the charging effect caused by the electron beam. As a conductive layer, ESpacer 300Z is applied over the resist to improve the effects of charging. The photonics lattice pattern is exposed using E-beam lithography (JBX-9300, JEOL). During the exposure process with the electron beam, uneven patterns can form due to scattering among electrons, depending on the shape and density of the pattern. To prevent this phenomenon, Proximity Effect Correction (PEC) functionality is applied. PEC refers to the shot modulation capability that allows different electron beam energy to be applied to different areas of the pattern, thereby forming a structure with uniform linewidths. The process conditions applied in this E-beam lithography process are an acceleration voltage of 100 kV, electron beam current of 300 pA, and Dose of 150 μC cm^−^².

The resist patterns formed by e-beam lithography are etched using an Inductively Coupled Plasma Dry Etcher (Multiplex ICP, Oxford). Given the variety in periods and linewidths of the designed structures, there is a concern for micro/macro loading effects during the dry etching process. The micro/macro loading effect refers to the phenomenon where etch rates vary depending on the linewidth and density of the pattern, respectively. This effect is related to the removal of by-products, and to improve the micro/macro loading effect, the process should be conducted at low pressure. However, continuously lowering the pressure to improve the loading effect can lead to inadequate plasma formation. The ICP mode, which applies RF Bias to both the top and bottom of the equipment, has a higher plasma density at low pressures compared to RIE mode. This allows for the improvement of micro/macro loading effects that can occur in dry etching equipment, resulting in structures with consistent periods and linewidths, and uniform etch depths. The conditions for this etching process involved injecting process gases SF6 at 45 sccm and O2 at 5 sccm, with a pressure of 7 mTorr, applied power of 2 kW, and a bias of 50 V.

**Figure S1. Step-by-step flow of the fabrication process.** **a** Align key pattern process for E-beam lithography. **b** Fabrication process of topological guided-mode resonance structures.
